# Automated segmentation and length measurement of metacarpal and phalangeal bones for hand radiograph evaluation

**DOI:** 10.1038/s41598-026-69557-5

**Published:** 2026-09-02

**Authors:** Philip Gutberlet, Aron Kirchhoff, Eike Bolmer, Philipp Schmidt, Fabio Hellmann, Johannes Grün, Alexander Hustinx, Elisabeth André, Thomas Schultz, Klaus Mohnike, Peter Krawitz, Behnam Javanmardi

**Affiliations:** 1 Institute for Genomic Statistics and Bioinformatics, Universitätsklinikum Bonn, Baunscheidtstr. 17, 53113 Bonn, Germany; 2 Medical Faculty, Otto-Von-Guericke-University Magdeburg, Leipziger Straße 44, 39120 Magdeburg, Germany; 3 Human-Centered Artificial Intelligence, University of Augsburg, Universitaetsstrasse 6a, 86159 Augsburg, Bavaria Germany; 4 b-it and Computer Science Department, University of Bonn, Bonn, 53115 Germany; 5 Lamarr Institute for Machine Learning and Artificial Intelligence, Bonn, Germany

**Keywords:** Pediatric hand radiograph, Deep learning, Segmentation, Length measurement, Automation, Diagnosis, Anatomy, Health care, Medical research

## Abstract

Evaluating hand and wrist radiographs is essential in pediatric endocrinology and clinical genetics, particularly for the assessment of suspected skeletal anomalies. In this study, we present *Auto-Bone-Caliper*, an automated system for the segmentation and length measurement of metacarpal and phalangeal (M&P) bones, trained and evaluated on public datasets comprising both normal and dysmorphic cases. We first introduce InstanceSAM, a two-stage framework that detects and segments all 19 M&P bones in pediatric hand radiographs, achieving Dice scores of 98.7% for normal bones and 95.0% for dysmorphic bones. We further develop and evaluate three methods for bone-length estimation, identifying a *k*-means–based approach as the most accurate, with relative errors of 2.2% for normal bones and 4.5% for dysmorphic bones. Our automated pipeline, *Auto-Bone-Caliper*, integrates InstanceSAM with the *k*-means–based length-estimation method. To enable scale-independent downstream analyses, we derive relative bone-length measures from the automated measurements. Using these relative measures, we statistically compare measurements obtained using *Auto-Bone-Caliper* on an independent dataset with a healthy reference catalog of normal bone morphologies, observing a high level of agreement (Wasserstein-1 distance = 0.012). Finally, we demonstrate a potential clinical use case of *Auto-Bone-Caliper* by obtaining relative metacarpophalangeal pattern profiles for three genetic conditions, namely Turner syndrome, achondroplasia, and pseudohypoparathyroidism. Our results highlight the potential of the *Auto-Bone-Caliper* to streamline and standardize M&P length measurement, providing an objective and reproducible tool suitable for clinical application.

## Introduction

The evaluation of hand and wrist X-rays is a fundamental component in pediatric endocrinology and clinical genetics, particularly for assessing children with suspected skeletal dysplasias and other bone anomalies^1^. Beyond their traditional role in estimating bone age^2–4^, these radiographs provide critical insights into bone morphology, growth plate architecture, and ossification patterns that can help identify or differentiate a wide spectrum of skeletal disorders^5^. In children with disproportionate short stature, deformities of the long bones, or abnormal bone density, detailed analysis of the hand X-ray can reveal diagnostic features such as metaphyseal irregularities, epiphyseal dysgenesis, and abnormal carpal ossification sequences characteristic of specific dysplasias (e.g., achondroplasia, pseudoachondroplasia, or metaphyseal chondrodysplasia)^6^. Moreover, hand radiographs assist in distinguishing endocrine causes of growth disturbance from structural or genetic bone disorders, guiding clinicians toward appropriate hormonal, genetic, or metabolic testing. The importance of hand X-rays in diagnosing skeletal dysplasias arises from their ability to capture the fine detail of ossification centers and cortical bone modeling–making them indispensable for both initial evaluation and longitudinal monitoring of children with abnormal skeletal development^5^. In particular, the lengths and proportions of individual metacarpal and phalangeal bones (hereafter referred to as M&P bones) are frequently altered across a wide range of bone disorders, and their detailed assessment can greatly aid in diagnosis^7–9^.

For example, metacarpophalangeal pattern profile (MCPP) analysis (which is the analysis of length and proportions of the M&P bones on a hand X-ray) have been shown to be a useful tool for diagnosing diastrophic dysplasia^10^, Sotos syndrome^11^, dyschondrosteosis, Turner syndrome, and hypochondroplasia^12,13^. A notable effort was done by A. K. Poznanski who analyzed and published M&P length measurements for healthy individuals and for over 100 disorders^8,14^.

However, manually measuring the 19 M&P bones in a hand radiograph is a laborious and time-consuming process that is also susceptible to human error. An automated method for quantifying M&P bone lengths in pediatric hand radiographs could therefore provide substantial clinical benefit by improving efficiency, consistency, and diagnostic accuracy. In this study, we introduce a novel automated approach designed specifically for this purpose.

From an image analysis perspective, accurate bone segmentation is a prerequisite for reliable length measurement. Several studies have explored segmentation of individual hand bones to enable quantitative skeletal analysis. Early model-based methods demonstrated feasibility using anatomical priors^15–17^. Later studies addressed pediatric and clinical contexts using increasingly advanced segmentation techniques. Phalangeal regions and epiphyseal/metaphyseal areas in children were segmented for growth assessment using image enhancement and boundary detection algorithms^18^, while active shape models were also applied for individual bones^19,20^. More recently, deep learning–based methods have enabled automated bone-level segmentation: fully automatic networks have been developed for phalanges segmentation in bone age analysis, and convolutional neural network (CNN) frameworks have been used to segment metacarpals and phalanges in arthritis and bone age studies^21–25^. On the length measurement topic, current studies have focused on the metacarpal bones^26,27^, while the phalangeal bones have received little to no consideration.

However, existing studies on hand-bone segmentation and measurement have primarily focused on cohorts with normal skeletal morphology, without evaluating model performance on dysmorphic bones–a limitation likely driven by the scarcity of such data.

**Fig. 1:**
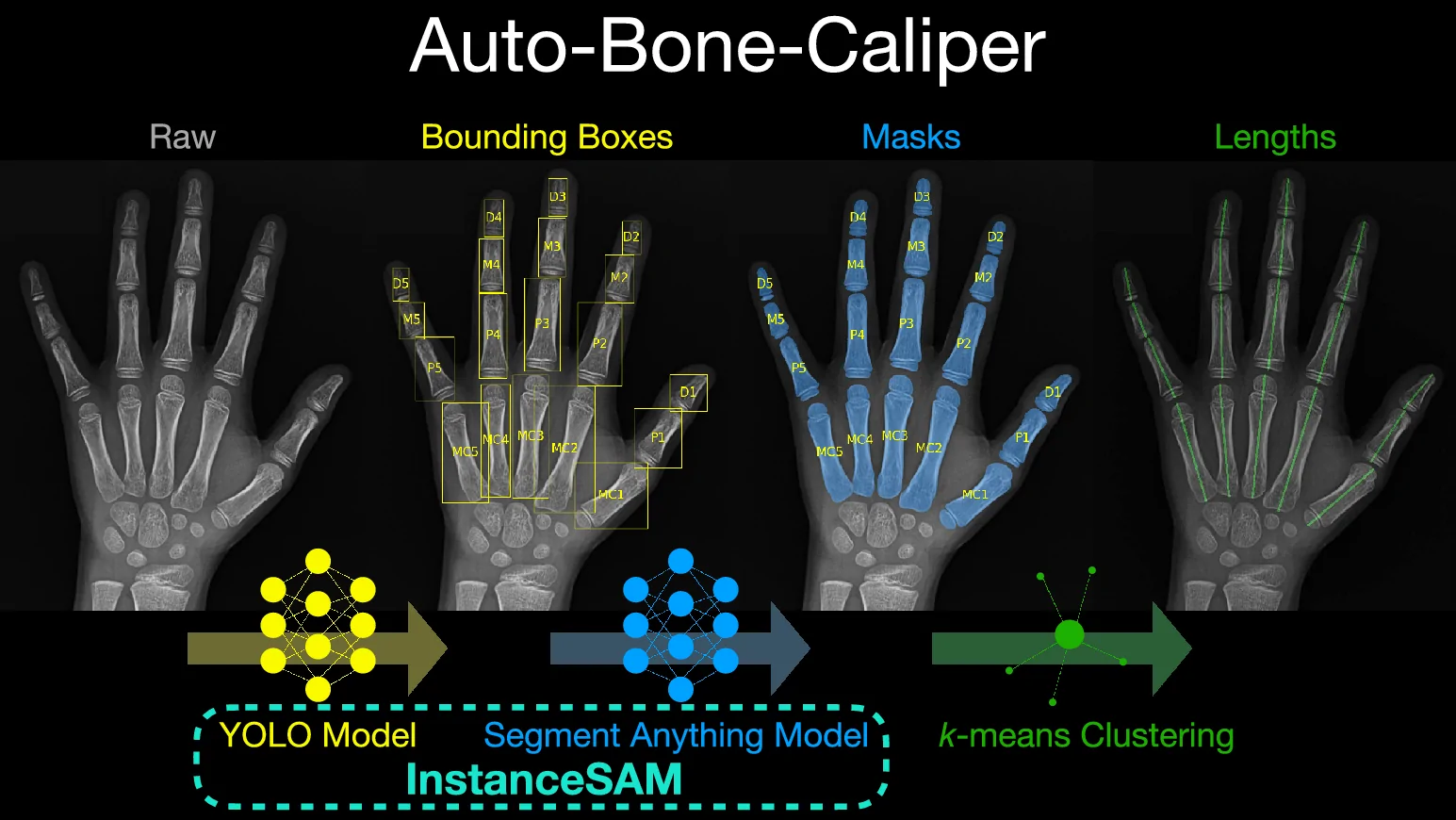
A schematic of our system, *Auto-Bone-Caliper*, for automatic instance segmentation and length measurements of the M&P bones. The raw radiograph is from the RSNA dataset. *MC* Metacarpal bone, *P* Proximal phalanx, *M* Middle phalanx, *D* Distal phalanx.

In this work, we introduce a new pipeline for automated segmentation and length measurement of the 19 M&P bones and assess its performance on normal and also dysmorphic hand radiographs that are frequently observed in genetic and endocrine bone disorders. Our proposed approach provides a step toward quantitative and objective evaluation of hand radiographs, with potential to enhance clinical assessment in these complex patient populations. Figure 1 shows a schematic of our automatic system which we call *Auto-Bone-Caliper*.

## Materials and methods

### Data collection

We used three public image data sources in this study.

#### RSNA bone age dataset

The primary dataset used in this work is from the Radiological Society of North America (RSNA) Pediatric Bone Age Challenge 2017^28^. This public dataset contains over 14,000 high-resolution pediatric hand X-rays with bone age and sex information^29^. The majority of these X-rays belong to individuals with normal hand bone morphology. From the RSNA dataset, we selected a subset of 550 X-rays with an equal number of male and female subjects. By randomly selecting images from sex-specific bins, the subset covers the entire range of bone ages. The radiographs were visually inspected to ensure quality and normal bone morphology. We used hand X-rays from the RSNA for the development of our segmentation model.

#### GestaltMatcher database

We obtained hand X-rays of patients with skeletal conditions from the GestaltMatcher Database (GMDB)^30,31^. GMDB is a collection of curated medical imaging of genetic syndromes as a resource for clinicians and researchers. From the GMDB, we used 50 hand X-rays from 12 different conditions, namely Turner syndrome (n=13), achondroplasia (n=10), pseudohypoparathyroidism (n=10), Proteus (mild phenotype, n=4), Ollier (n=3), McCune-Albright syndrome (n=3), Down syndrome (n=2), Marfan syndrome (n=1), Noonan syndrome (n=1), Hypochondroplasia (n=1), Leri-Weil syndrome (n=1), and Pseudopseudohypoparathyroidism (n=1). We used hand X-rays from the GMDB for testing the performance of our segmentation and length measurement methods on dysmorphic bones.

#### Digital hand atlas dataset

As an additional test set of normal morphology bones for testing our length measurement approach, we used the publicly available Los Angeles Digital Hand Atlas (DHA) dataset^32^, which contains 1,390 radiographs acquired between 1997 and 2008 at the Children’s Hospital Los Angeles, USA. The DHA dataset contains radiographs from individuals of four different ancestry groups: Caucasian, Asian, African American, and Hispanic. Since both the RSNA dataset and the data of Poznanski (1984)^8^ (see below) are primarily based on children of European ancestry, we restricted our analysis to the corresponding ancestry group in the DHA dataset, comprising 330 hand X-rays.

**Fig. 2:**
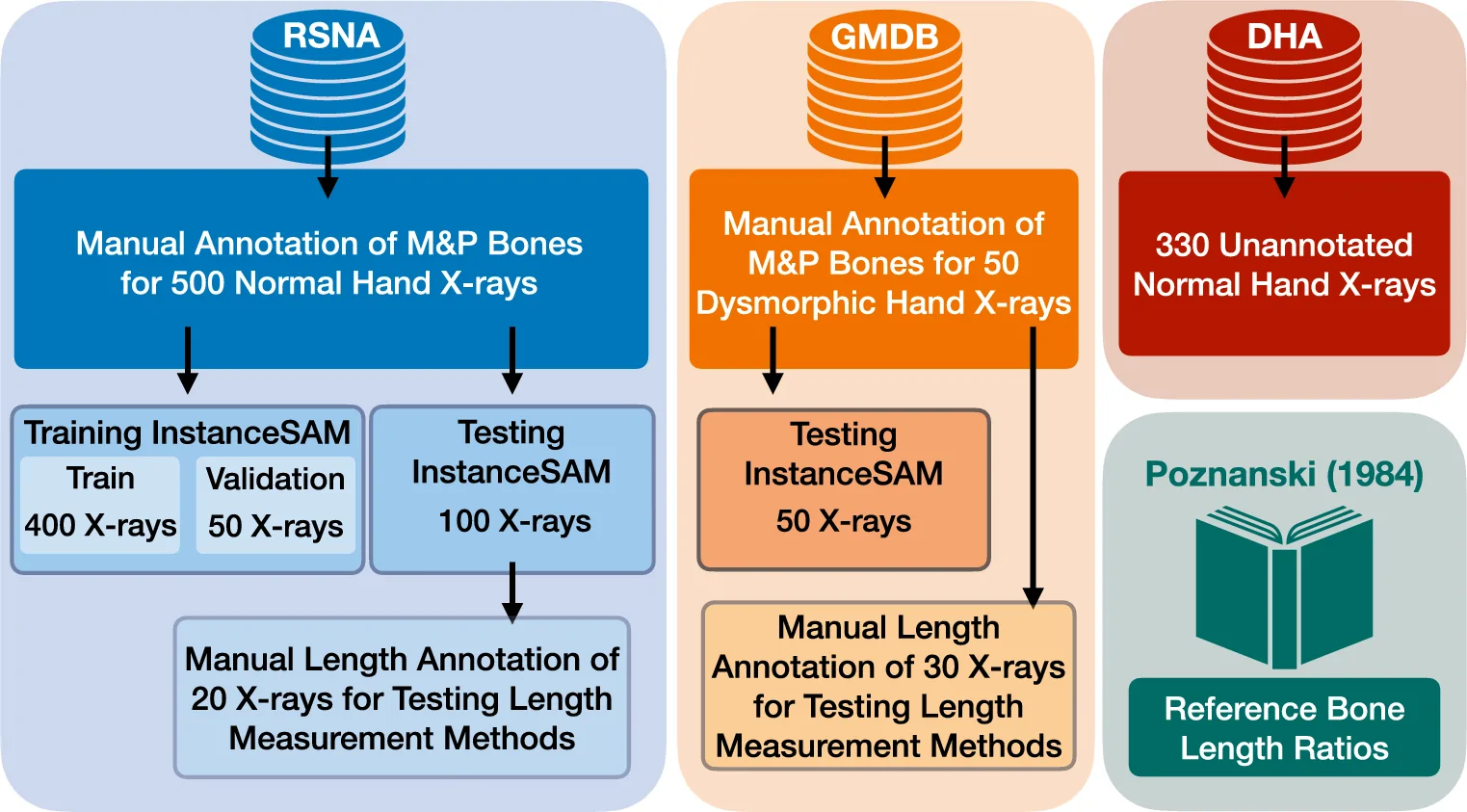
The datasets used in this study. They are all publicly avilable. RSNA: Radiological Society of North America, *GMDB* GestaltMatcher Database, *DHA* Digital Hand Atlas.

In addition to these image data sets, we also used the reference M&P bone length measurements from Poznanski (1984)^8^. These references contain mean and standard deviation values of bone lengths for different age ranges and for both males and females from a healthy cohort. It also contains ratios of the lengths of the 19 M&P bones which enables a scale-independent analysis. We used these reference ratios to test the performance of *Auto-Bone-Caliper* when applied on an independent healthy cohort (from the DHA dataset). Figure 2 summarizes the datasets used in this study.

### Data preparation

#### Bone mask annotations

The image datasets mentioned above provide neither bone annotations nor bone masks. Consequently, we used the Labelbox Annotation Tool (https://www.labelbox.com) to manually annotate segmentation masks and labels for the 19 M&P bones in all 550 hand X-rays from the RSNA and 50 hand X-rays from the GMDB, resulting in 11,400 bone masks with labels.

#### Manual length measurements

We manually measured the lengths of the M&P bones, using Labelbox, in 20 normal and 30 dysmorphic hand radiographs (10 cases each for Turner syndrome, achondroplasia, and pseudohypoparathyroidism). The manual length measurements were done by a clinician-scientist (P.S.) following clinical guidelines^7^ guided by a pediatric endocrinologist with more than 40 years of clinical experience (K.M.). These annotations are the ground truth for testing our automated length measurement methods (see below). Throughout this paper, we follow the standard anatomic naming approach. Finger numbers start with the thumb (1) and go to the little finger (5).

### Deep learning model for bone segmentation

We develop a two-stage approach for automatic instance segmentation of the 19 M&P bones consisting of the YOLO v9 model^33^ followed by the Segment Anything Model v1 (hereafter, SAM)^34^. First, we fine-tuned a YOLO v9 model with pre-trained weights (COCO2017) for 50 epochs with data augmentations (horizontal flipping, random scaling, random brightness). For this purpose, we used 400 RSNA hand X-rays for training, 50 for validation, and 100 for test. This model was trained to predict the bounding boxes and class labels for each of the 19 M&P bones. The model’s performance was measured on the average precision with an intersection over union (IoU) threshold set to 0.5. Subsequently, the outputs from the fine-tuned YOLO model, i.e. the predicted bounding boxes, are used as input for SAM, to give it a cue to the location of the bone to segment. We used the ViT-H SAM model pre-trained on the SA-1B dataset. We refer to the combination of our fine-tuned YOLO model and SAM as InstanceSAM (Fig. 1) and use the Dice score as the metric for quantifying its performance.

### Algorithms for bone length measurements

**Fig. 3:**
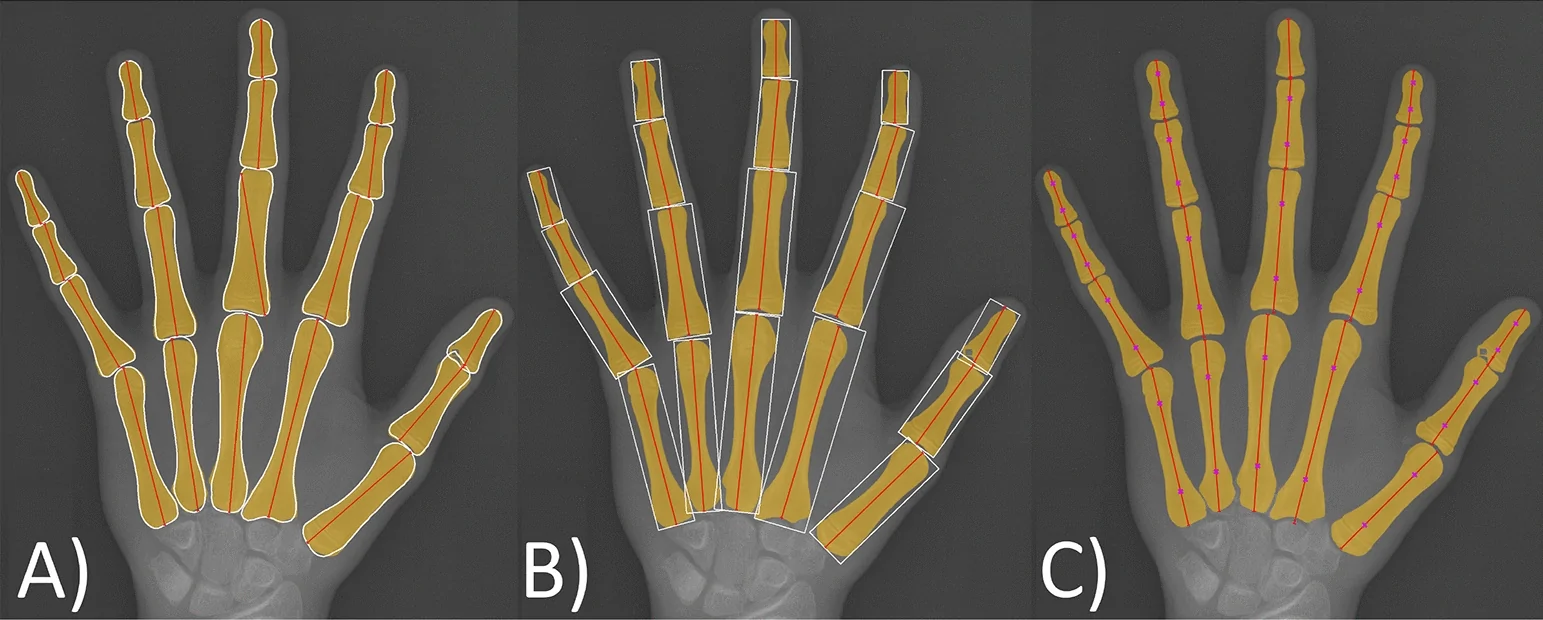
Length measurements for a hand with normal bone morphology. The red lines show the lengths, the bounding boxes and contours are shown in white, and the cluster centers are pink crosses. The methods used are: (**A**) Statistical shape model, (**B**) Bounding boxes, and (**C**) *k*-means.

Once InstanceSAM predicts the segmentation masks for each bone, the next challenge is accurately calculating bone lengths. While this may seem straightforward, adhering to clinical measurement standards is essential. There are two main challenges in measuring bone length: identifying the outline and defining the bone axis along which the length is measured. Simple methods, such as identifying the longest line within the mask, are insufficient, as this would yield a diagonal line. Clinical guidelines require length to be measured along the bone’s main axis, terminating at its outermost points. We developed three algorithms to determine the main axis and length of the bone. These methods are described below and depicted in Fig. 3.

#### Statistical shape model

In this method, we build and use 19 Statistical Shape Models (SSM), one per bone type, to place 100 points around each bone mask. These 100 points are placed evenly along the contour of each bone mask. Those contours are then aligned through iterative affine transformations and mean shape matching. We then perform Procrustes analysis with Principal Component Analysis (PCA) retaining *95%* of the variance to get a model for the bone shapes. The same PCA transformations are then applied to contours of new segmentation masks, to ensure consistent point placement around the segmentation mask. The main axis on the test set is defined by the pair of points that minimizes the axis error in the training set.

#### Bounding box

This method applies a function to compute a bounding box enclosing the bone best. The minimum-area bounding rectangle of a given set of points is determined by identifying the smallest enclosing rectangle that fully contains the shape while allowing arbitrary rotation. This process involves analyzing the convex hull of the points and applying the Rotating Calipers algorithm to find the rectangle with the least area. The resulting rectangle is characterized by its center coordinates, dimensions, and orientation angle. The centers of the bounding box’s top, bottom, left, and right edges are then determined based on its orientation. Finally, the height and width of the bone are measured by calculating the Euclidean distances between these centers.

#### *k - means*

In this method, we use *k*-means to find the main bone axis by splitting the pixels in the segmentation mask of each bone into two clusters. The idea is that one cluster will form at the proximal end, the other at the distal end of the bone mask. We use *k*-means clustering with *k=2*, giving us the main axis by connecting the cluster centers. To find the length, we then rotate the segmentation mask to align the main axis with the y-axis of the image. The length can then be extracted from the extent of the bone in y-Direction.

We evaluate each bone length estimation method using the manual annotations (see Section 2.2.2) by calculating the average relative length and axis angle errors.

Given that the image scales across different imaging devices can be different, or the physical size of each pixel might be absent in some images, we need to work with dimensionless (scale-independent) values, i.e., length ratios, for the following analyses.

### Comparison with poznanski reference values

As mentioned in Section 2.1, Poznanski (1984)^8^ provides sex- and age-specific M&P bone length ratios and their standard deviations for a healthy cohort. As a conservative test of *Auto-Bone-Caliper*, we consider Poznanski (1984) values as healthy references and compare our M&P length ratio measurement on a subset of the DHA dataset which was not part of our training process.

For this purpose, we compute pairwise bone length ratios derived from segmented bone measurements. The 19 M&P bones in each radiograph yield *N = 171* unique pairwise ratios $R_{x,y}$ defined as

1 $$\begin{aligned} R_{x,y} = \frac{l_{x}}{l_{y}}, \end{aligned}$$

where $l_{x}$ and $l_{y}$ denote the lengths of bones *x* and *y* in pixels, respectively.

We then quantify the relative difference of our automated measurement for a healthy cohort from the reference values of Poznanski $R^{\text {ref}}_{x,y}$^8^ via

2 $$\begin{aligned} \Delta R_{x,y} = \frac{(R_{x,y} - R^{\text {ref}}_{x,y})}{R^{\text {ref}}_{x,y}}. \end{aligned}$$

We then analyze the distribution of these relative differences by measuring its mean and standard deviation and comparing it with the distribution from Poznanski (1984) using Wasserstein-1 distance.

**Fig. 4:**
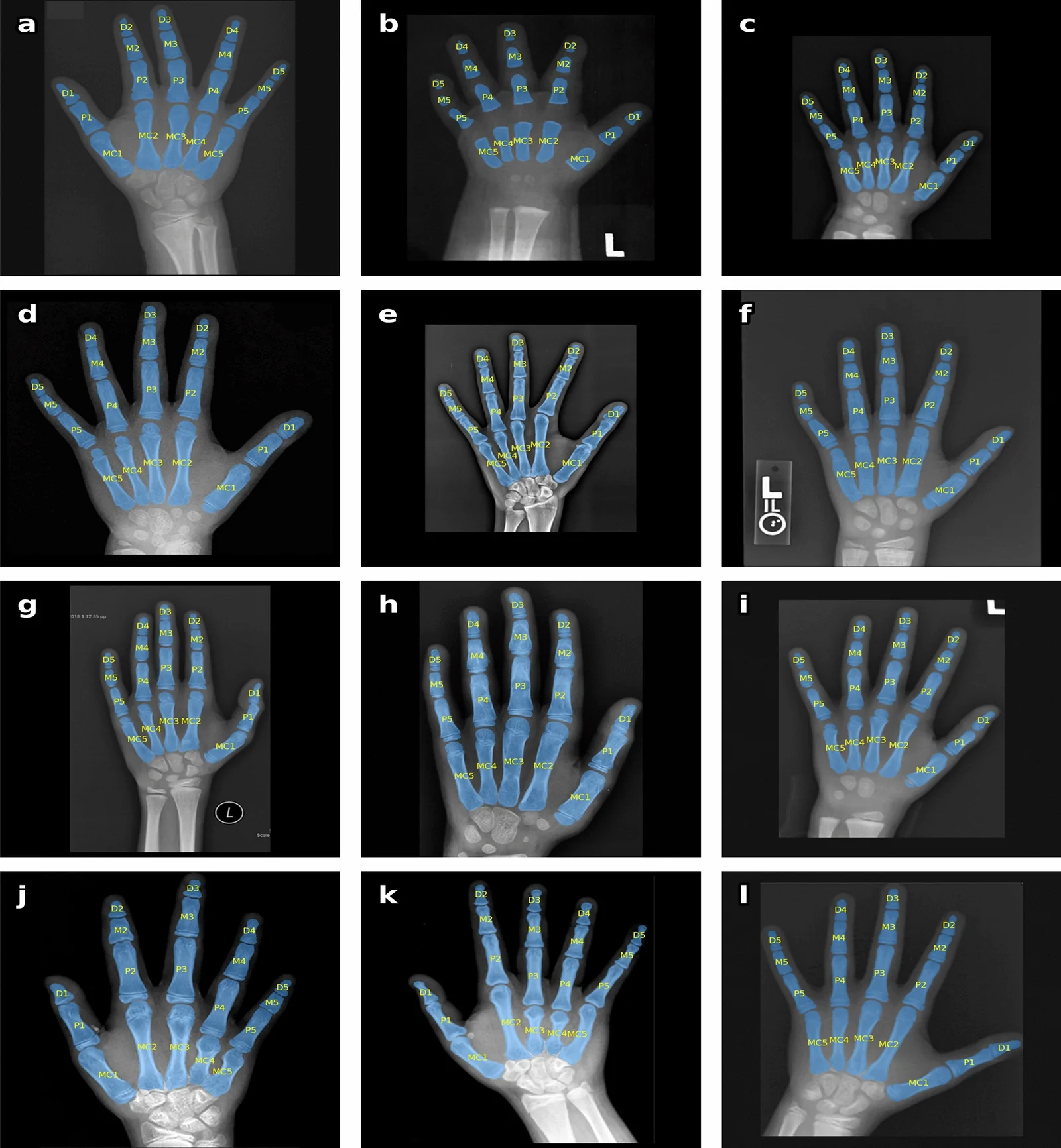
Examples of performance of InstanceSAM on dysmorphic hand X-rays. (**a**) achondroplasia, (**b**) Down syndrome, (**c**) hypochondroplasia, (**d**) Leri-Weill syndrome, (**e**) Marfan syndrome, (**f**) McCune–Albright syndrome, (**g**) Noonan Syndrome, (**h**) Ollier disease, (**i**) Proteus syndrome, (**j**) pseudohypoparathyroidism, (**k**) pseudopseudohypoparathyroidism, (**l**) Turner syndrome. The raw images are obtained via the GMDB^31^, note the variations in scales and quality. *MC* Metacarpal bone, *P* Proximal phalanx, *M* Middle phalanx, *D* Distal phalanx.

### Metacarpophalangeal pattern profile

To obtain the MCPPs, we first define the relative bone length $r_{m,x}$ of bone *x* in radiograph *m* as:

3 $$\begin{aligned} r_{m,x} = \frac{l_{m,x}}{\sum _{i=1}^{19} l_{m,i}} \end{aligned}$$

where $l_{m,x}$ is the bone length in pixels. This essentially normalizes the length of each M&P bone in a hand with the sum of the lengths of all the 19 M&P bones in that hand, creating scale-independent values. Then we use the mean bone length ratio $\overline{r}^s_x$ across all radiographs of a sample *s*. This is then used to infer MCPPs that can be visualized by plotting the z-score:

4 $$\begin{aligned} z^{s}_{x} = \frac{\overline{r}^{s}_{x} - \overline{r}^\text {ref}_x}{\sigma ^\text {ref}_x} \end{aligned}$$

using the mean $\overline{r}^\text {ref}_x$ and standard deviation $\sigma _x$ from a reference healthy cohort.

## Results

### Bone segmentation

The mean Dice score of InstanceSAM for normal hands was *98.7%* and for dysmorphic bones in the GMDB test set was *95.0%*. In Table 1 we also list the average DICE score for each of the 19 bones in the normal and dysmorphic cohorts. For the normal cohort, the mean DICE score for all bones is above *98.0%* and the general trend is a decreasing DICE score for smaller bones. This trend shows itself more drastically in the dysmorphic cohort with the lowest DICE score, *84.3%*, belonging to the smallest bone in the hand i.e. the distal phalanx of the 5th finger.

Examples of the performance of InstanceSAM segmentation on dysmorphic bones of 12 different disorders in the GMDB test set are shown in Fig. 4.

**Table 1:** Performance of InstanceSAM. The Mean Dice score for all 19 bones and for each bone type is listed for both normal and dysmorphic hand radiographs.

Bone	Normal [%]	Dysmorphic [%]
All 19 Bones	98.7	95.0
Metacarpal 1	99.0	97.1
Metacarpal 2	98.6	97.4
Metacarpal 3	98.3	97.5
Metacarpal 4	98.3	97.1
Metacarpal 5	98.8	97.1
Proximal Phalanx 1	99.0	95.6
Proximal Phalanx 2	99.3	97.4
Proximal Phalanx 3	99.4	97.8
Proximal Phalanx 4	99.3	97.5
Proximal Phalanx 5	99.2	95.7
Middle Phalanx 2	98.0	96.1
Middle Phalanx 3	98.4	94.5
Middle Phalanx 4	98.7	94.1
Middle Phalanx 5	98.3	94.9
Distal Phalanx 1	98.8	94.6
Distal Phalanx 2	98.3	90.7
Distal Phalanx 3	98.5	94.3
Distal Phalanx 4	98.5	91.1
Distal Phalanx 5	98.2	84.3

### Bone length prediction

Performances of the three automatic bone length measurement methods are provided in Table 2. The result reveals notable differences between normal and dysmorphic groups. Across all methods, the relative error is consistently lower for normal hand radiographs compared to dysmorphic ones. The *k*-means–based method achieved the best performance in both axis detection and length prediction, yielding errors of only 2.2% and $1.6^{\circ }$, respectively, for normal hands, and 4.5% and $2.9^{\circ }$, respectively, for dysmorphic hands. Therefore, we choose the *k*-means approach as the length measurement method of our *Auto-Bone-Caliper* and use it for all the subsequent analyses.

**Table 2:** Average relative length and axis angle errors in percentage *[%]* and degrees [$^{\circ }$], respectively, for the three automatic length measurement methods.

Method	Normal (n=20)		Dysmorphic (n=30)		Total (n=50)	
	Length	Angle	Length	Angle	Length	Angle
Statistical shape model	3.1%	$5.6^{\circ }$	6.4%	$10.3^{\circ }$	5.1%	$8.4^{\circ }$
Bounding Box	3.4%	$3.5^{\circ }$	5.6%	$5.1^{\circ }$	5.7%	$4.6^{\circ }$
*k*-means	2.2%	$1.6^{\circ }$	4.5%	$2.9^{\circ }$	3.6%	$2.4^{\circ }$

### Comparison with Poznanski’s reference values

For the length-ratio comparisons, the age and sex of the cases must be taken into account. Poznanski (1984) provides bone length ratios for age groups of 1, 4, 9, and 18 years. By setting N=10 as the minimum number of images required for this comparison, we restrict our analysis to cases aged 4, 9, and 18 years in the DHA dataset, comprising 10, 15, and 20 radiographs, respectively.

**Fig. 5:**
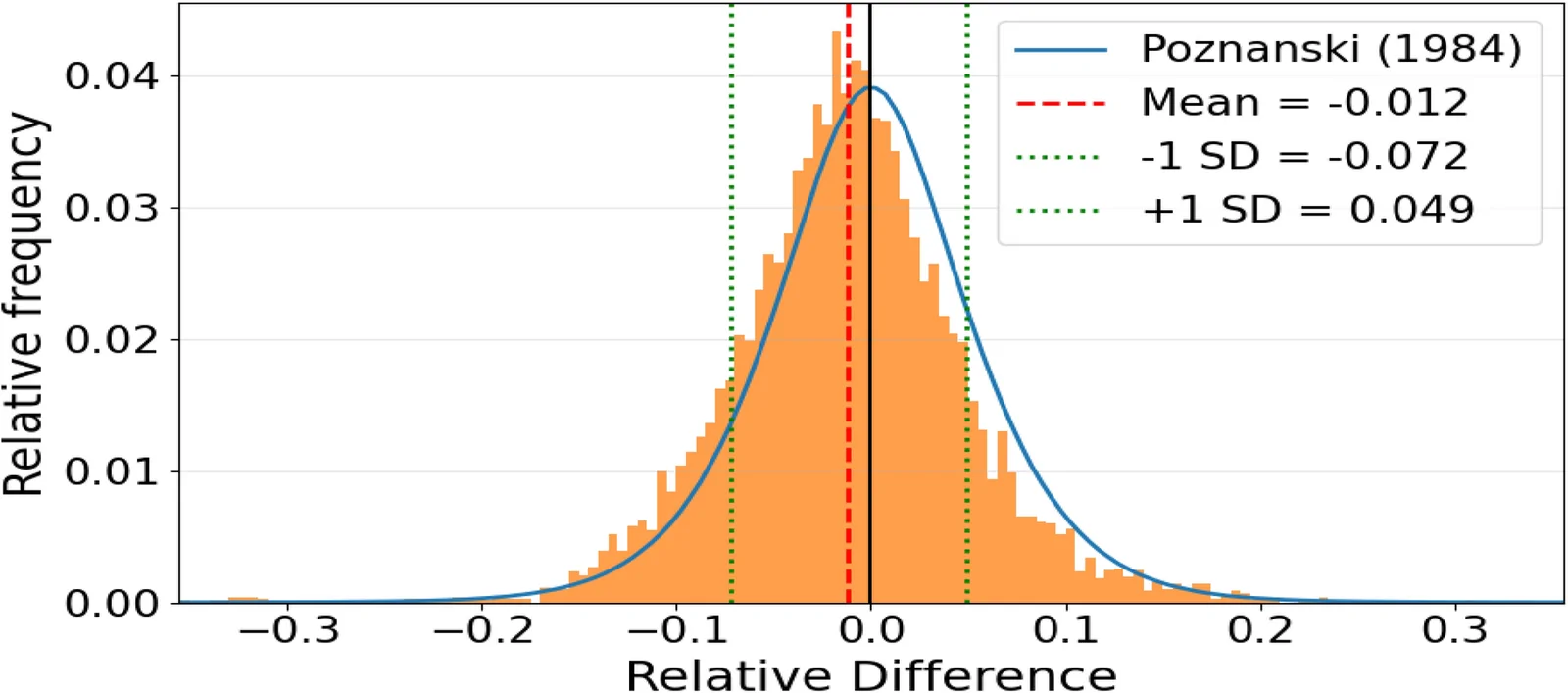
Distribution of relative differences (Eq. 2) between predicted bone-length ratios on 45 radiographs (ages 4, 9, and 18) from the DHA dataset and Poznanski (1984)^8^ reference values.

Figure 5 presents the distribution of relative differences (Eq. 2) for the 45 radiographs. For comparison, we also show a reconstructed distribution corresponding to Poznanski (1984), derived from the reported standard deviations under the assumption of Gaussianity. Treating Poznanski (1984) as the reference, its relative difference with respect to itself has zero mean and a standard deviation of *± 0.0593*.

The distribution of relative differences obtained via our *Auto-Bone-Caliper* closely matches that of Poznanski (1984), with a mean of $-0.012^{+0.049}_{-0.072}$, corresponding to a standard deviation of *± 0.060*. The Wasserstein-1 distance between the two distributions is 0.012, consistent with their nearly identical dispersions.

### Metacarpophalangeal pattern profile

**Fig. 6:**
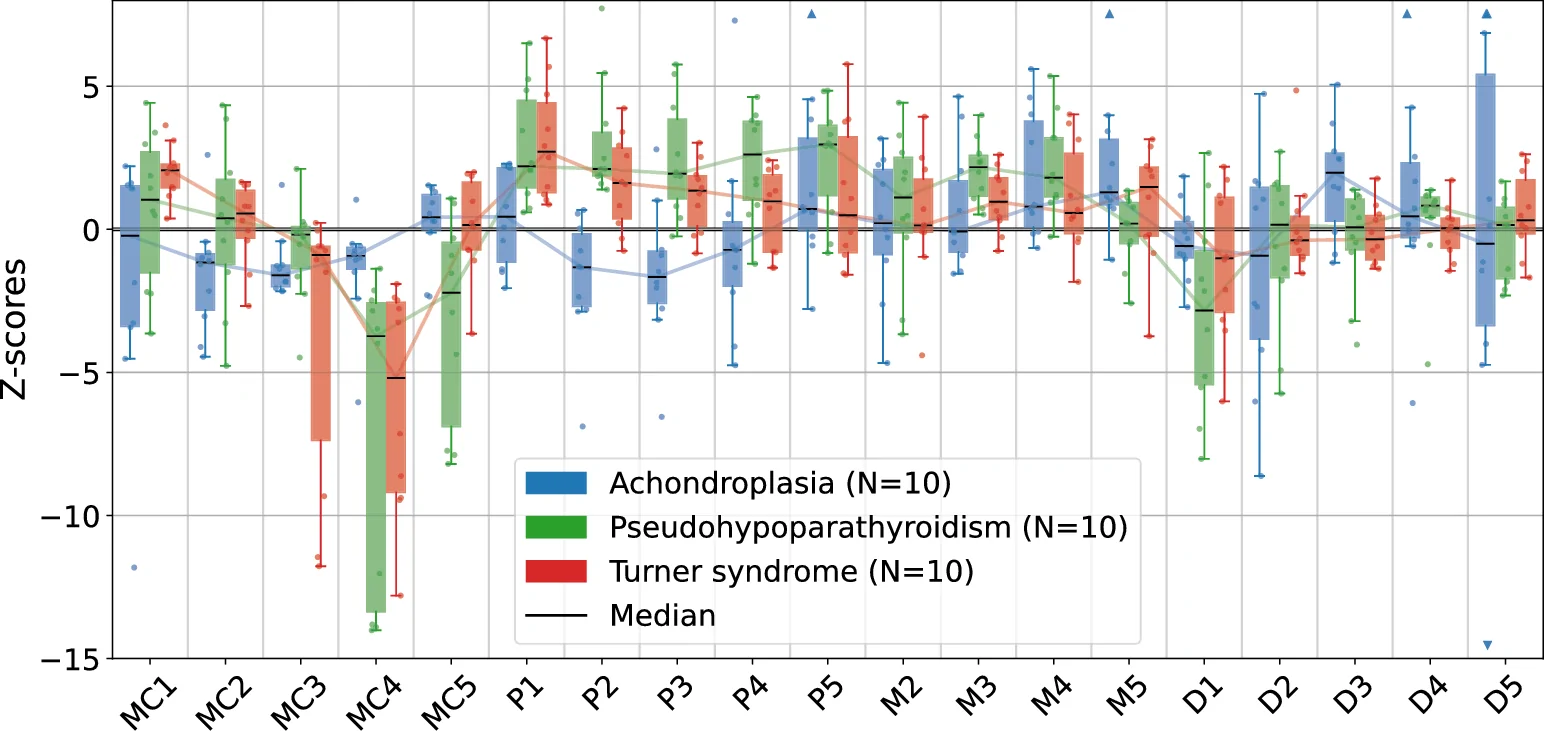
The MCPPs from the relative bone lengths for three dysmorphic groups: Achondroplasia, Pseudohypoparathyroidism, and Turner syndrome (*N=10* patients per group), with the DHA dataset (male and female) as reference (Z-score *=0*). For each bone, a box plot shows the distribution of Z-scores across the patients of the group (box: interquartile range (IQR); line: median; whiskers: 1.5*×*IQR), with the individual patients overlaid as points; triangles at the plot edge mark points outside the y-axis range. The colored lines connect the per-bone medians of each group.

As an example of the use-case of our *Auto-Bone-Caliper* we show in Fig. 6 MCPPs of three different dysmorphic groups i.e. Turner syndrome, achondroplasia, and pseudohypoparathyroidism. The results from the previous section indicate a high general concordance between the reference values of Poznanski (1984)^8^ and healthy cases in DHA. Therefore, we now take our automatic measurements from all the 330 DHA radiographs as reference (Z-score=0, see Eq. 4) for building MCPPs.

The profiles show characteristic differences. Fore example, the most common abnormalities for pseudohypoparathyroidism, a (relative) shortening of MC4 and MC5^35^, are visible through negative z-scores for these bones. For achondroplasia, absolute brachydactyly cannot be demonstrated, as we could only use relative bone lengths (Eq. (3)). The same holds for Turner syndrome; however, the characteristic (relative) shortening of MC4 characteristic of this syndrome^35^ is clearly visible. However, it should be emphasized, that these MCPPs were derived from relatively small cohorts and should therefore be considered illustrative examples rather than diagnostic reference profiles.

## Discussion

Our segmentation model, InstanceSAM, achieves a substantially higher Dice score than those reported in previous studies^24,25^, both of which target bone age assessment. Owing to differences in experimental settings and datasets, a direct quantitative comparison is challenging. In the two-stage approach presented in^25^, which was applied to data from a local hospital in China, hand bones are first extracted using the OSA-YOLOv5 network and subsequently segmented with GRU-UNet. Although that study also includes segmentation of the radius and ulna, only the M&P bones of the 1 st, 3rd, and 5th digits are segmented. Their average Dice score for the M&P bones is around 95%. In^24^, the authors propose a lightweight encoder–decoder semantic segmentation model with a multi-scale attention block, achieving a Dice score of 93.9% on the RSNA and MURA^36^ datasets. It is important to note, however, that^24^ additionally segments the radius, ulna, and carpal bones, whereas our work focuses exclusively on the M&P bones for which length-based measurements can be reliably defined and evaluated.

At the time of writing, we are not aware of any segmentation studies specifically addressing dysmorphic hand bones, precluding direct comparison. The lower performance of our segmentation model on dysmorphic bones compared to normal ones can be attributed to two main factors: (1) the model was trained exclusively on normal bone morphology, and (2) the hand X-rays in GMDB are sourced from figures in publications and therefore exhibit substantially reduced image quality both in resolution and intensity. The particularly low Dice score for the fifth distal phalanx may additionally reflect its small size and the difficulty of delineating the proximal epiphysis, especially in younger children with incomplete ossification. Because this bone occupies relatively few pixels, small absolute segmentation errors can also produce a larger relative reduction in Dice score. Representative suboptimal cases are shown in Supplementary Figs. S1 to S5. Nevertheless, the model’s ability to generalize to dysmorphic cases despite being trained exclusively on unaffected morphology is encouraging and supports the feasibility of using such models for downstream quantitative analyses.

Estimating bone length from a segmentation mask is not trivial, because the longest distance between two contour points may follow a diagonal and therefore does not necessarily correspond to the clinically relevant longitudinal bone axis. Established approaches to analogous shape-measurement problems include statistical shape models, principal-axis or bounding-box–based geometric methods, and skeleton- or centerline-based measurements^15–17,19,20^. In this study, we investigated three representative strategies: a statistical shape model that uses anatomically consistent contour points, an oriented minimum-area bounding box, and a *k*-means–based method that estimates the longitudinal axis from the spatial distribution of mask pixels. The latter provides a simple data-driven approximation of the proximal–distal axis without requiring a predefined bone template or explicit skeletonization.

Regarding the length-measurement methods, our results show a clear advantage of the *k*-means–based approach–for both normal and dysmorphic bones–over the SSM and bounding-box techniques, which exhibit comparable performance. Accurate detection of the bone axis is essential for reliable length estimation, and the axis-prediction error of the *k*-means method is approximately half that of the other two approaches. For this reason, we selected *k*-means as the length-measurement component of *Auto-Bone-Caliper*.

The novelty of *Auto-Bone-Caliper* lies not only in its segmentation performance, but in the integration of instance segmentation and clinically oriented length measurement for all 19 metacarpal and phalangeal bones within a single automated pipeline. Previous automated bone-length approaches have primarily focused on the metacarpals or on restricted subsets of hand bones^26,27^, while recent hand-bone segmentation methods have generally been developed for bone-age assessment and have not provided complete length measurements for all M&P bones^24,25^. Phalangeal length measurement and automated extraction of complete MCPP have therefore received considerably less attention. In addition, existing methods have predominantly been developed and evaluated on radiographs with unaffected morphology^24–27^. Our approach extends this work by evaluating the complete segmentation and measurement pipeline on both unaffected and dysmorphic bones and by deriving scale-independent relative measurements that can be used to generate MCPPs and compare them with established reference data^8^. The combination of full 19-bone coverage, evaluation in dysmorphic cases, and downstream relative-length analysis therefore represents the principal methodological contribution of this study.

A key result of this study is the evaluation of our *Auto-Bone-Caliper* on normal hand radiographs from the DHA dataset–which was not used during training–using the reference values provided in the Poznanski (1984)^8^ catalog. Two considerations are important here. First, the Poznanski reference values were derived from manual measurements performed with caliper on physical hand radiographs in the 1970s and 1980 s, rather than digital images. Second, there is more than a decade between the data collection of the DHA dataset (1997–2008) and the acquisition and publication of the RSNA dataset, during which imaging protocols may have changed substantially. For these reasons, we regard this evaluation to be a conservative one.

The MCPPs for achondroplasia, pseudohypoparathyroidism, and Turner syndrome (Fig. 6) illustrate the potential of the system to automatically extract MCPPs for comparison with established reference data. It should be emphasized that these MCPPs were derived from relatively small cohorts and should therefore be regarded as illustrative examples rather than diagnostic reference profiles. In a recent study^37^, has highlighted the renewed value of MCPP analysis for the evaluation of short stature and skeletal dysplasias by providing reference values for a Brazilian cohort.

The intended clinical role of *Auto-Bone-Caliper* is to provide automated and standardized measurements of metacarpal and phalangeal bone lengths rather than to generate an autonomous diagnosis. Such quantitative phenotyping may complement the clinical and genetic evaluation of children with short stature, as recent international guidelines recommend hand and wrist radiography not only for bone-age assessment but also for identifying anatomical variants that may guide genetic investigation^38^. In a potential clinical workflow, a hand radiograph would be processed automatically to segment the 19 M&P bones and derive absolute or relative length measurements. The clinician would review the segmentation and measurements, correct them if required, and interpret the resulting profile together with the patient’s age, sex, clinical findings, and other diagnostic information. The system may therefore serve as a quantitative decision-support tool by reducing the time and variability associated with manual measurements and by facilitating comparison with established reference profiles. Prospective studies are required to determine how reliably this workflow can be integrated into routine practice and whether it improves efficiency, reproducibility, or clinical decision-making.

### Limitations

This study has several limitations. First, the dysmorphic test cohort was relatively small and included only 50 radiographs from 12 conditions, with several disorders represented by only a few cases. In addition, InstanceSAM was trained exclusively on radiographs with normal bone morphology, which may limit its performance in patients with severe or previously unseen skeletal abnormalities. Including representative dysmorphic cases during training or fine-tuning would likely improve segmentation performance, although the greater morphological variability within these conditions would remain an important challenge. Such an extension would require the collection and manual annotation of a sufficiently large and diverse set of dysmorphic radiographs, which is challenging given the rarity and morphological heterogeneity of these conditions. However, the aim of the present study was specifically to determine whether a model trained on unaffected individuals could generalize sufficiently to support downstream analyses in patients with dysmorphic bone morphology. The accuracy of the automated length measurements was also evaluated on a limited reference set of 50 radiographs. Moreover, the manual measurements were performed by a single clinician-scientist under the guidance of an experienced pediatric endocrinologist, and inter-rater variability was not formally assessed. Although independent datasets were used for evaluation, the system has not yet been prospectively validated on routine clinical radiographs acquired across multiple institutions, imaging devices, and patient populations. The dysmorphic radiographs obtained from GMDB were also heterogeneous in resolution, contrast, scaling, and overall image quality, as many were derived from published figures. Furthermore, the comparison with healthy reference values relied on the historical Poznanski catalog, which was derived several decades ago using physical radiographs and may not fully reflect contemporary imaging protocols, current populations, or secular changes in growth. This comparison was additionally restricted to selected age groups and predominantly European-ancestry cohorts, limiting generalizability across ages and ancestries. Because reliable pixel-spacing information was not consistently available, the analyses were based on scale-independent bone-length ratios rather than absolute measurements, preventing the assessment of generalized absolute shortening such as brachydactyly. In a clinical deployment setting, however, physical pixel-spacing information is typically available from the imaging metadata and could be incorporated to derive absolute bone-length measurements. Finally, the disease-specific MCPPs were based on small cohorts and should be regarded as illustrative rather than diagnostic reference profiles.

## Conclusion

This study introduces *Auto-Bone-Caliper*, an automated system for segmentation and length measurement of the M&P bones. We first present InstanceSAM, a two-stage segmentation pipeline that detects 19 M&P bones in pediatric hand X-rays using a fine-tuned YOLO model, followed by segmentation with SAM. Although InstanceSAM was trained exclusively on normal hand radiographs, it generalizes successfully to dysmorphic bones from patients with various bone disorders.

We then designed and evaluated three bone-length measurement methods: statistical shape modeling, bounding box estimation, and a *k*-means–based approach. The *k*-means method demonstrated superior accuracy and was therefore selected as the length-measurement component of our *Auto-Bone-Caliper*.

To assess the reliability of *Auto-Bone-Caliper*, we derived scale-independent relative bone-length measures and compared bone-length ratios obtained from normal hand radiographs in the independent DHA dataset with the reference values for healthy individuals reported in the Poznanski (1984)^8^ catalog. The close agreement between the two supports the validity of *Auto-Bone-Caliper* for downstream analyses.

Bone-length analysis in hand radiographs, such as in MCPP analysis, is known to aid in the diagnosis and evaluation of patients with various skeletal disorders; however, the burden of manual measurements has limited its routine clinical use. Automated solutions such as *Auto-Bone-Caliper* have the potential to streamline and standardize the measurement process, enabling objective and scalable quantitative assessment for clinical evaluation and longitudinal monitoring. The resulting measurements may support, but should not independently determine, the diagnosis of patients with skeletal abnormalities.

## Supplementary Information

## Data Availability

The scripts generated during the current study are available in the Auto-Bone-Caliper repository, https://github.com/bone2gene/Auto-Bone-Caliper. The data annotations generated during the current study are available from the corresponding author on reasonable request.
