# Supplementary material for: Automated segmentation and length measurement of metacarpal and phalangeal bones for hand radiograph evaluation
Source: Sci Rep. 2026 Sep 2;16:27514. doi: 10.1038/s41598-026-69557-5 (PMC13538351; doi:10.1038/s41598-026-69557-5)
Supplement: Supplementary file 1 [file 41598_2026_69557_MOESM1_ESM.pdf]

Supplementary Figures of the manuscript

# Automated Segmentation and Length Measurement of Metacarpal and Phalangeal Bones for Hand Radiograph Evaluation

Philip Gutberlet[1], Aron Kirchhoff[1], Eike Bolmer[1], Philipp Schmidt[2], Fabio Hellmann[3], Johannes Grün[4], Alexander Hustinx[1], Elisabeth André[3], Thomas Schultz[4,5], Klaus Mohnike[2], Peter Krawitz[1], and Behnam Javanmardi[1]*

[1] Institute for Genomic Statistics and Bioinformatics, Universitätsklinikum Bonn, Baunscheidtstr. 17, 53113 Bonn, Germany

[2] Medical Faculty, Otto von Guericke University Magdeburg, Magdeburg, Germany

[3] Human-Centered Artificial Intelligence, University of Augsburg, Universitätsstrasse 6a, 86159 Augsburg, Bavaria, Germany

[4] Bonn-Aachen International Center for Information Technology (b-it) and Department of Computer Science, University of Bonn, 53115 Bonn, Germany

[5] Lamarr Institute for Machine Learning and Artificial Intelligence, Germany

*Corresponding author: Behnam Javanmardi
Email: bjav@uni-bonn.de

In this Supplementary Information document, we present examples of suboptimal segmentation outputs across several bone disorders and briefly describe the likely causes of these deviations.

Turner

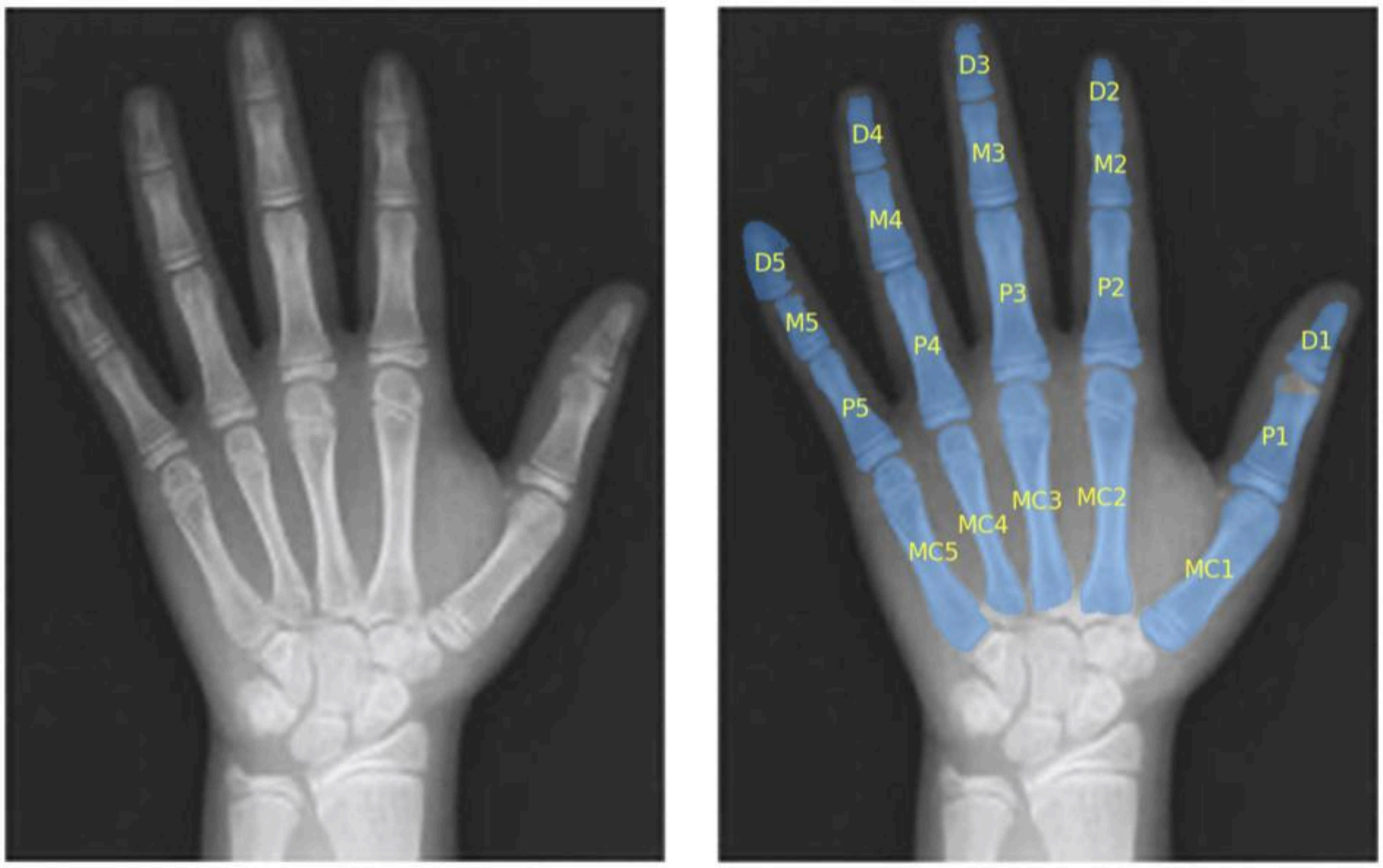


Supplementary Figure S1: A case of Turner. The D5 shows low contrast relative to the surrounding tissue, causing the segmentation to extend beyond the bone boundaries.

Ollier disease

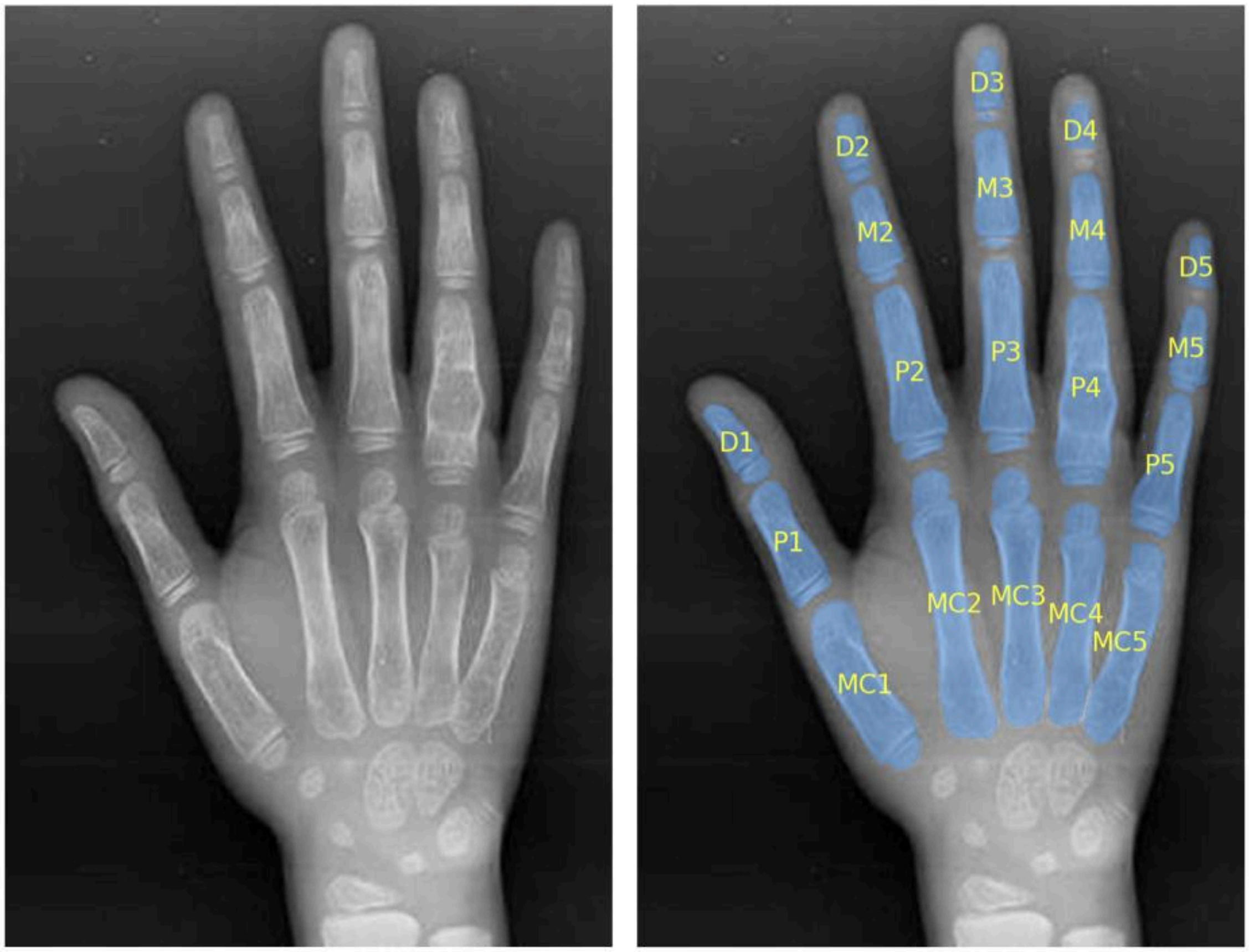


Supplementary Figure S2: A case of Ollier disease. The epiphysis of the D5 bone is missed by the model.

## Turner

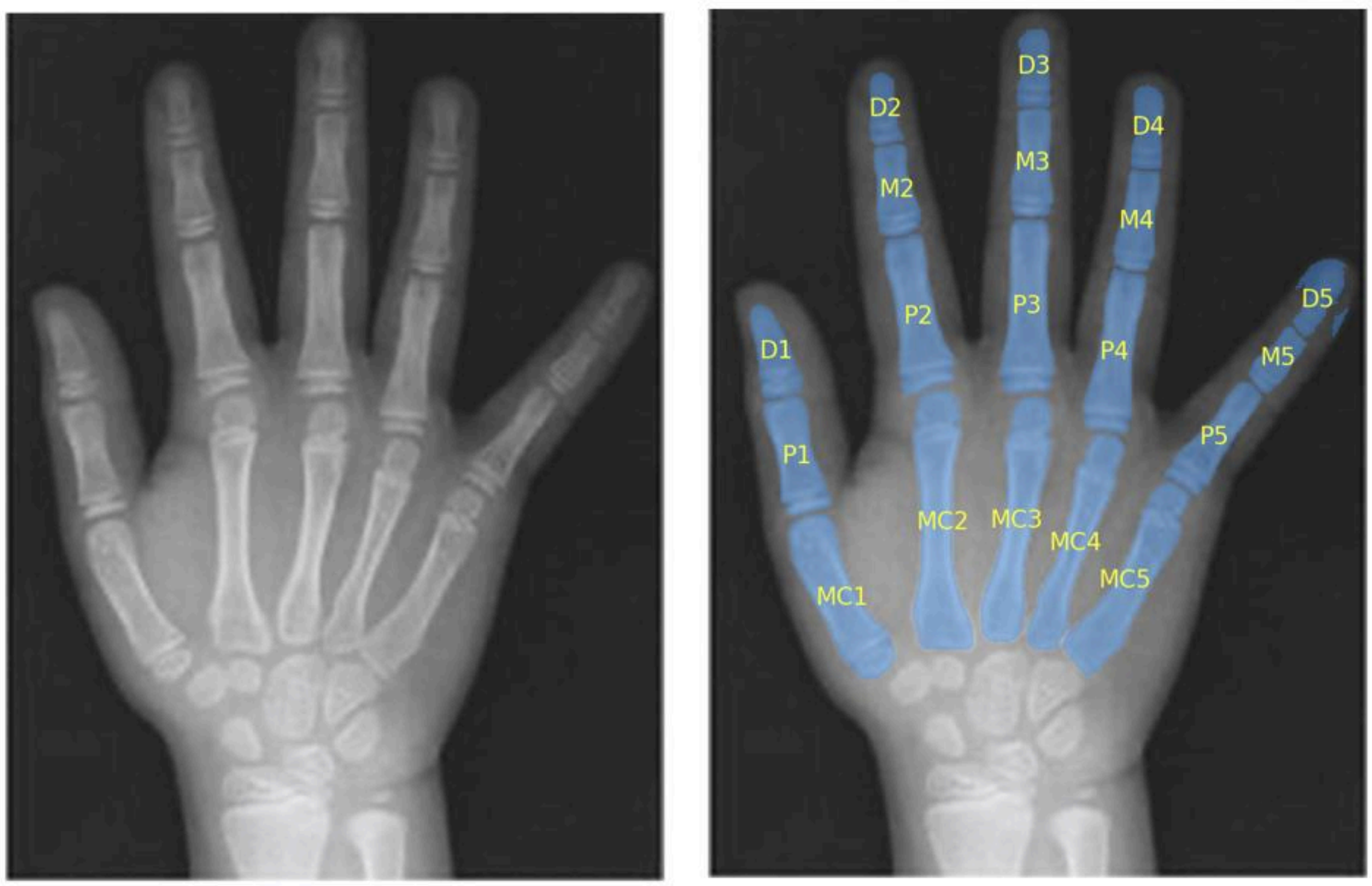


Supplementary Figure S3: A case of Turner syndrome. The D5 shows low contrast relative to the surrounding tissue, causing the segmentation to extend beyond the bone boundaries.

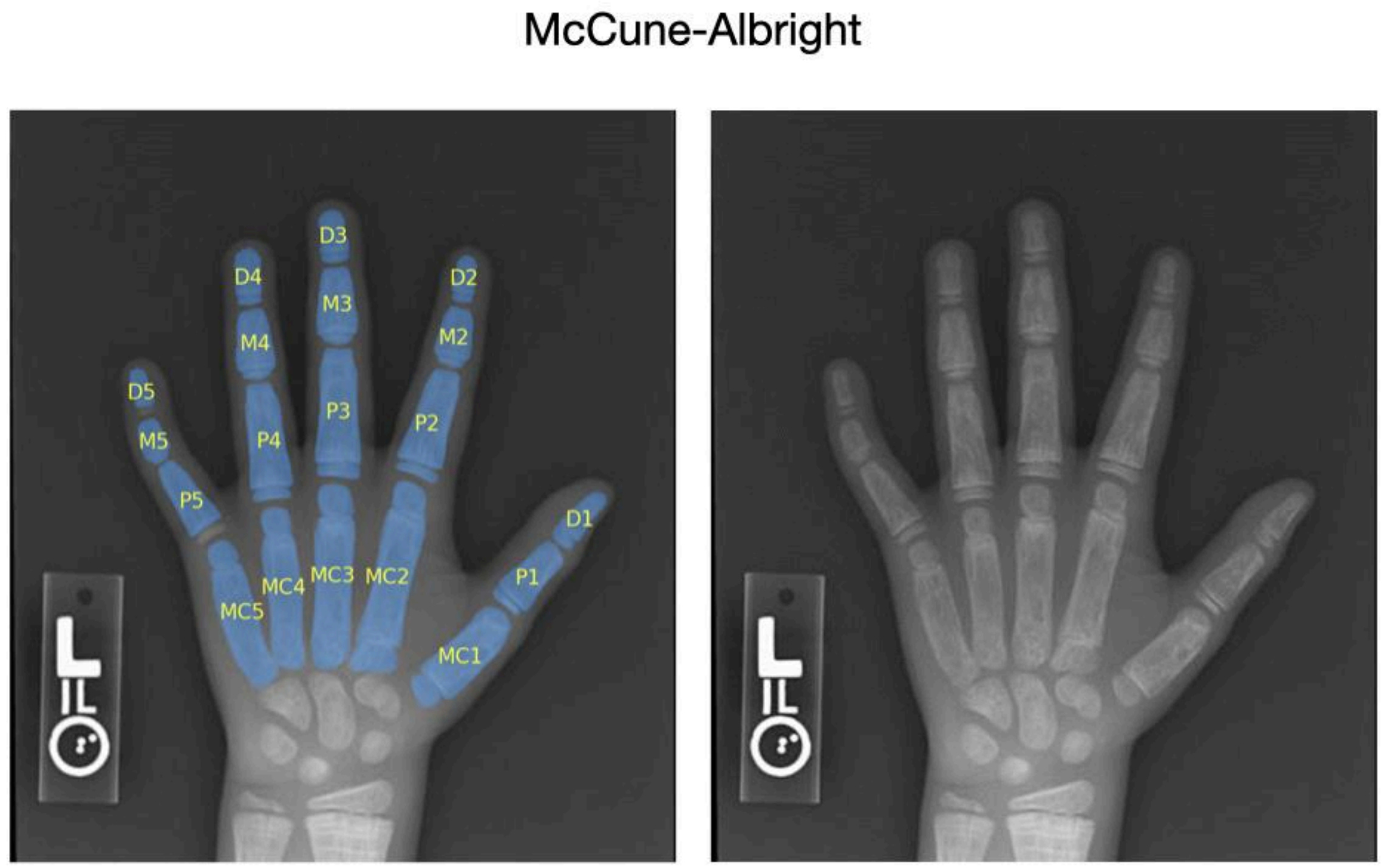


Supplementary Figure S4: A case of McCune-Albright syndrome. The epiphysis of the D5 bone is missed by the model.

## Proteus

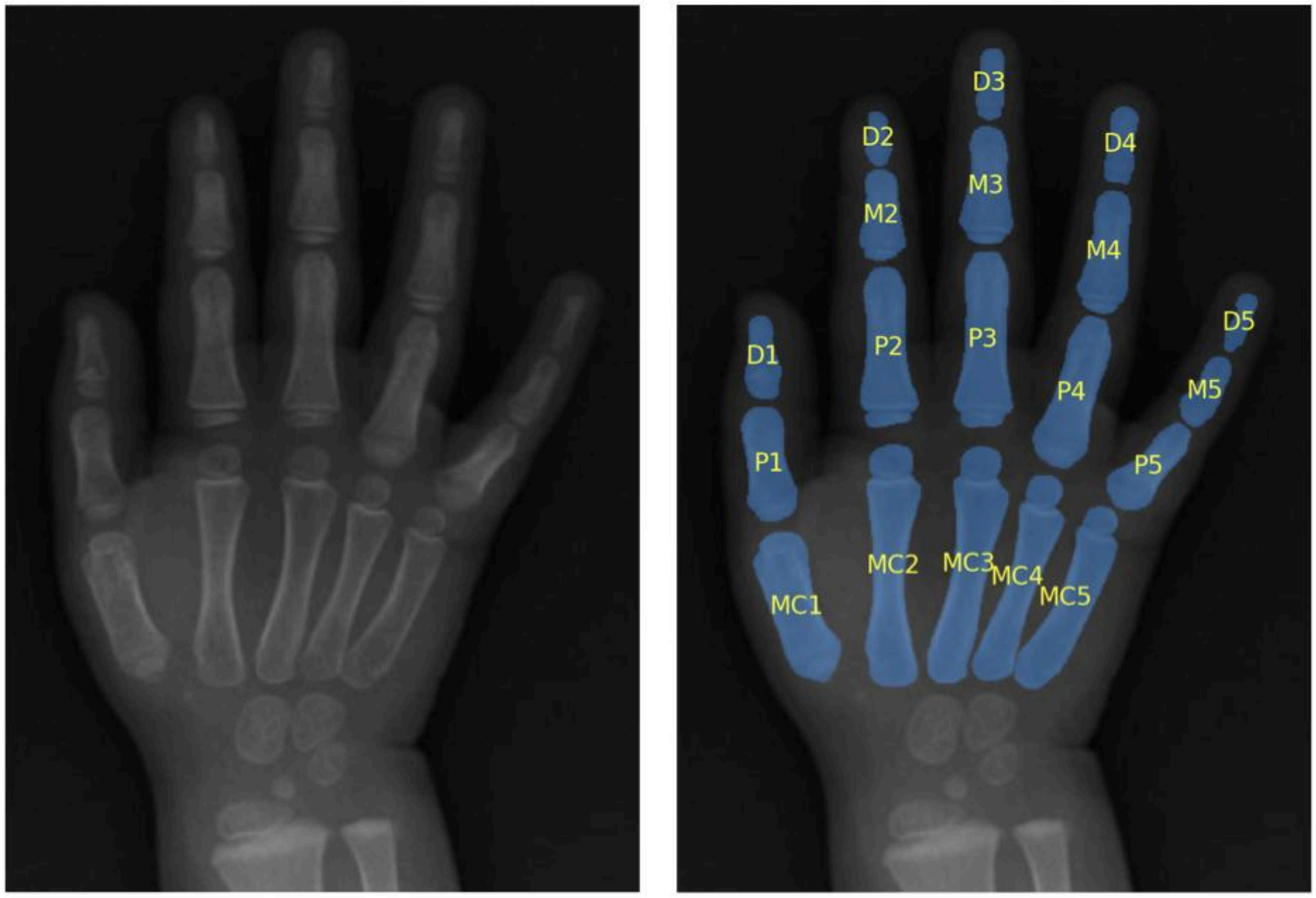


Supplementary Figure S5: A case of Proteus syndrome. The D5 shows low contrast relative to the surrounding tissue, causing the segmentation to extend beyond the bone boundaries.
